# Caesarean Delivery and Subsequent Stillbirth or Miscarriage: Systematic Review and Meta-Analysis

**DOI:** 10.1371/journal.pone.0054588

**Published:** 2013-01-23

**Authors:** Sinéad M. O’Neill, Patricia M. Kearney, Louise C. Kenny, Ali S. Khashan, Tine B. Henriksen, Jennifer E. Lutomski, Richard A. Greene

**Affiliations:** 1 National Perinatal Epidemiology Centre, Anu Research Centre, Department of Obstetrics and Gynaecology, Cork University Maternity Hospital, Wilton, Cork, Ireland; 2 Department of Epidemiology and Public Health, University College Cork, Cork, Ireland; 3 Anu Research Centre, Department of Obstetrics and Gynaecology, Cork University Maternity Hospital, Wilton, Cork, Ireland; 4 Perinatal Epidemiology Research Unit, Department of Paediatrics, Aarhus University Hospital, Aarhus, Denmark; The University of Adelaide, Australia

## Abstract

**Objective:**

To compare the risk of stillbirth and miscarriage in a subsequent pregnancy in women with a previous Caesarean or vaginal delivery.

**Design:**

Systematic review of the published literature including seven databases: CINAHL; the Cochrane library; Embase; Medline; PubMed; SCOPUS and Web of Knowledge from 1945 until November 11^th^ 2011, using a detailed search-strategy and cross-checking of reference lists.

**Study Selection:**

Cohort, case-control and cross-sectional studies examining the association between previous Caesarean section and subsequent stillbirth or miscarriage risk. Two assessors screened titles to identify eligible studies, using a standardised data abstraction form and assessed study quality.

**Data synthesis:**

11 articles were included for stillbirth, totalling 1,961,829 pregnancies and 7,308 events. Eight eligible articles were included for miscarriage, totalling 147,017 pregnancies and 12,682 events. Pooled estimates across the stillbirth studies were obtained using random-effect models. Among women with a previous Caesarean an increase in odds of 1.23 [95% CI 1.08, 1.40] for stillbirth was yielded. Subgroup analyses including unexplained stillbirths yielded an OR of 1.47 [95% CI 1.20, 1.80], an OR of 2.11 [95% CI 1.16, 3.84] for explained stillbirths and an OR of 1.27 [95% CI 0.95, 1.70] for antepartum stillbirths. Only one study reported adjusted estimates in the miscarriage review, therefore results are presented individually.

**Conclusions:**

Given the recent revision of the National Institute for Health and Clinical Excellence guidelines (NICE), providing women the right to request a Caesarean, it is essential to establish whether mode of delivery has an association with subsequent risk of stillbirth or miscarriage. Overall, compared to vaginal delivery, the pooled estimates suggest that Caesarean delivery may increase the risk of stillbirth by 23%. Results for the miscarriage review were inconsistent and lack of adjustment for confounding was a major limitation. Higher methodological quality research is required to reliably assess the risk of miscarriage in subsequent pregnancies.

## Introduction

A recent review reported that in high-income countries, one in every 200 pregnant women reaching 22 weeks gestation and beyond will have a stillborn baby [1]. The UK has one of the highest stillbirth rates of high-income countries with only France and Australia ranking higher [1]. There were 4,100 stillbirths reported in the UK in 2009, a rate of 3.5 per 1,000 births, or 11 stillbirths every day. Globally, more than three million pregnancies result in a stillbirth annually, the majority arising in developing countries [2]. Reform of the classification of stillbirths is urgently needed, particularly the criteria for recording the cause of death and other vital information [3]. No one classification system is commonly accepted, with varying definitions of stillbirth used by investigators, countries, health organisations, and classification schemes [4]. Stillbirths can be defined according to gestational age at birth typically into early stillbirths (20–28 weeks gestation) and late stillbirths (>28 weeks) [5]. Additionally stillbirths are classified into antepartum (death occurring before the onset of labour) or intrapartum (death during or after labour) [6]. However, the primary method for classification of stillbirth is according to the apparent cause or associated obstetric disorders.

Stillbirths were first classified using the Aberdeen classification system based solely on available clinical information [7]. This was followed by the British perinatal mortality survey in the 1950’s which used autopsy data [4], [8] and most recently by the Wigglesworth criteria which were developed in the 1980’s and are the most widely used criteria to date [9]. In Australia stillbirth is defined as any fetus born weighing more than 400 grams, or more than 20 weeks in gestation [10]. In the United Kingdom, the definition of stillbirth is any fetus stillborn after 24 weeks gestation [11]. Furthermore, many systems include both stillbirths and neonatal deaths. These variations in the lower gestational age limit the ability to compare findings from different studies. Earlier classification systems included only a few categories (congenital malformations, immaturity, and asphyxia) whilst more recent systems have tried to include more hierarchical information on fetal growth, placental changes, and maternal disorders [5], [12]. Therefore, the use of conflicting classification systems, of which there are more than thirty in existence (with an additional twelve modifications of such systems) [12], may result in a deficit of essential information and a large proportion of unexplained stillbirths. In keeping with this, the contribution of unexplained stillbirths has been reported to be as high as 70% [13]. For this reason, researchers and clinicians have strived to better classify stillbirths according to the aetiology and models of causation for more than two decades [14].

Important known causes of stillbirth common to developed and developing countries include placental insufficiency with fetal growth restriction [15], infection, pre-eclampsia, congenital abnormalities, placental abruption and umbilical cord accidents [11], [16]–[19]. In addition, short inter-pregnancy intervals, prior stillbirths and a history of adverse pregnancy outcomes have all been associated with increases in stillbirth risk in developing countries [20]. Several risk factors for stillbirth have been identified, including primiparity, advanced maternal age, high BMI, maternal conditions such as pre-eclampsia, diabetes and hypertension, low educational attainment and socioeconomic status [1], [21], although the exact cause of stillbirth is often unknown [1], [22].

Spontaneous miscarriage (before 24 weeks gestation) is the most common early pregnancy complication with miscarriage rates ranging between 10% and 15% of recognised pregnancies [23], [24]. Some studies report that approximately one in five pregnancies will end in a miscarriage [24]–[26]. This number would be even greater if very early pregnancy losses or missed miscarriages are included, with rates of over 33% reported [23], [26], [27]. Similar to stillbirth, there have been many definitions for miscarriage but the most accepted and widely used is the World Health Organization’s (WHO) definition developed in 1977 [28]. Following this, miscarriage is defined as “the expulsion or extraction from its mother of an embryo or fetus weighing 500 g or less”. Miscarriage can be further sub-classified into early miscarriage (6±12 weeks gestation) or late miscarriage (12±24 weeks gestation) [29]. Therefore, variability in the definitions of miscarriage and stillbirth may affect the precision of recordings in registration systems, community and hospital surveys, together with those for measurement and comparison [30].

Chromosomal aneuploidies are reported to account for about 50–70% of miscarriages [31], [32] (the commonest being monosomy X and trisomy 16), followed by thrombophilia [33]. Risk factors for miscarriage include caffeine consumption, alcohol and drug use, previous induced abortions and uterine defects [24]. Stillbirth and miscarriage share several risk factors including smoking [34]–[38], advanced maternal age [39]–[43], history of pregnancy loss [5], [32], [44], [45], and body mass index (BMI) [1], [29], [46]–[48]. History of Caesarean delivery has been implicated as a risk factor for both stillbirth [1] and miscarriage [49]–[51], however, to date, evidence is conflicting [52], [53]. The underlying mechanisms for an association between Caesarean delivery and stillbirth and miscarriage are unclear but may be related to placental abnormalities. However, often these adverse events occur with no obvious underlying cause [1].

Understanding potential long-term adverse effects associated with Caesarean delivery is essential given the exponential rise in Caesarean rates over the past three decades [54]–[56]. Caesarean rates currently range from over 25% in the UK [57] and 35% in the USA [58], [59], to over 40% in certain Latin American countries including Brazil, Chile and Argentina [60]. The National Institute for Health and Clinical Excellence (NICE) guidelines [61], which were recently updated and give women the choice to request a Caesarean delivery without medical necessity, may lead to an increase in already high Caesarean rates. The aim of this systematic review is to examine the association between Caesarean delivery and subsequent risk of stillbirth and miscarriage.

## Materials and Methods

### Primary Objective

The main objective of this systematic review and meta-analysis is to synthesise the available published literature to date on the relationship between prior Caesarean delivery and risk of stillbirth or miscarriage in the subsequent delivery and to report an estimate of the increase in odds of stillbirth or miscarriage following a Caesarean delivery.

### Primary Outcomes

The outcomes of interest in this review are stillbirth (explained, unexplained, antepartum or intrapartum) and miscarriage following a Caesarean delivery.

### Search Strategy

In accordance with the preferred reporting items for systematic reviews and meta-analyses statement (PRISMA) [62], we conducted a systematic review and meta-analysis of the published literature (without language or date restrictions). We selected potentially eligible studies published between 1945 up until November 11^th^ 2011, from CINAHL, the Cochrane Library, Embase, Medline, PubMed, SCOPUS and Web of Knowledge databases with the following combined text and Medical Subject Headings (MeSH) including the exposure, outcome and study design (#Caesarean section AND #stillbirth OR #miscarriage AND #Case-control OR #Cohort study OR #Cross-sectional (Appendix S1). We supplemented our electronic search by cross-checking the reference lists of all identified studies. We included studies which published quantitative estimates of the association between mode of delivery and stillbirth or miscarriage. Eligibility criteria for inclusion in the meta-analysis included:

1. Data were from an original study (i.e. no review articles, editorials or commentaries).
2. Cohort, cross-sectional or case-control studies, in which mode of delivery in the previous pregnancy was reported and stillbirth or miscarriage in the subsequent pregnancy were the outcomes of interest.
3. No strict definition of stillbirth or miscarriage was followed in the review. It was necessary only that there was a clear statement or understanding that “stillbirth” or “miscarriage” was the outcome of interest in each eligible study.
4. Reporting of relative risk (RR), odds ratio (OR) or hazard ratio (HR) (or adequate data in order to compute these parameters), of mode of delivery associated with stillbirth or miscarriage.

### Study and Data Collection Processes

Titles and abstracts of studies retrieved from the search strategy were reviewed using the appropriate inclusion and exclusion criteria. The full text article was obtained for all potentially eligible studies for further appraisal.

### Data Abstraction

Using a data abstraction form, two assessors (SMON, RAG) individually selected data on study design, year of study, mode of delivery, stillbirth, miscarriage and potential confounding variables including smoking, maternal age, history of miscarriage or stillbirth and BMI. Discrepancies in data abstraction between assessors were resolved through consensus.

### Statistical Analysis

Our principal analysis investigated the overall risk of stillbirth or miscarriage in women with previous Caesarean delivery versus previous vaginal delivery. Pooled estimates across studies were obtained by means of random-effect models. Studies were weighted according to an estimate of statistical size defined as the inverse of the variance of the OR. We generated a funnel plot of the overall OR and a standard error (SE) to assess publication bias for each primary outcome. For stillbirth, 10 out of the 11 eligible studies reported adjusted estimates and these are reported in the meta-analysis. Where data were presented in a way that could not be included in a meta-analysis, results of the studies are presented individually. For miscarriage, only one study out of the eight eligible studies reported adjusted estimates and therefore it was decided that a meta-analysis would not be appropriate and as a result the study estimates are presented individually.

#### Subgroup analyses

We estimated separate ORs for studies which reported an adjusted estimate for explained stillbirths, for unexplained stillbirths and for studies where cause of stillbirth was not specified or reported. In addition, we separately analysed studies including antepartum stillbirths only. Subgroup analyses by cause and timing of stillbirth is important in terms of confounding and estimating the role of unexplained stillbirths in any potential association.

#### Sensitivity analyses

We undertook two sensitivity analyses in the stillbirth meta-analysis. First, we estimated the pooled OR by study design (cohort, cross-sectional). This was considered important as various types of study designs may differ in methodological quality. For example, cohort studies would generally have a much larger sample size, thereby generating more statistical power and potentially less biased estimates compared to smaller case-control studies. Second, sensitivity analyses by parity (primiparous versus multiparous) were performed. This was important to assess the degree of confounding by number of previous pregnancies. Analyses were performed using SAS 9.2 (SAS Institute Inc., Cary, NC), and the meta-analysis was conducted using Review Manager version 5.1 software [63].

#### Statistical heterogeneity

Heterogeneity between studies was examined by assessing differences in study characteristics including study setting (country, origin), study design (case control, cohort, cross-sectional), sampling frame (institutional or population-based), and definition of the outcome measure used. We assessed the degree of variability amongst studies attributable to between-study heterogeneity with the I^2^ statistic. Thresholds for the interpretation of I^2^ as recommended by the Cochrane Handbook for Systematic Reviews were followed in this review [64]. An I^2^ value of 0% to 40% suggests heterogeneity might not be important; 30% to 60% represents moderate heterogeneity; 50% to 90% represents substantial heterogeneity; and 75% to 100% represents considerable heterogeneity. According to the Cochrane Handbook, the importance of the I^2^ value is dependent on the magnitude and direction of effects as well as the strength of the evidence for heterogeneity (Chi-squared test P-value, 95% confidence interval for I^2^).

#### Population Attributable Risk (PAR)

The population attributable risk (PAR) is the proportion of the incidence of disease in the population (exposed and unexposed) that is due to the exposure. The PAR of stillbirth is an estimate of the total number of cases of stillbirth in the population that can be attributed to a particular exposure, in this instance prior Caesarean delivery. The PAR was calculated according to a formula used by Last et al. [65] including the adjusted estimates; 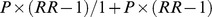, where *P* is the proportion of total population with the exposure. Population attributable risk percent (PAR %) which is the percent of the incidence of a disease in a population that is due to exposure was also calculated using the following formula: 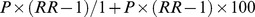. PAR and PAR% were calculated for all stillbirths in the total population as well as for unexplained stillbirths only.

### Quality Assessment

Two reviewers (SMON, RAG) using a quality assessment tool based on six different types of bias common in observational studies (selection, exposure, outcome, analytic, attrition and confounding) assessed the overall study quality. This bias classification tool has been described in detail elsewhere [66], [67]. Study bias was classified as minimal, low, moderate or high according to the degree of expected bias present for each of the six different types of bias and an overall likelihood of bias based on the total of the six different types of bias measured was reported.

## Results

**Figure 1:**
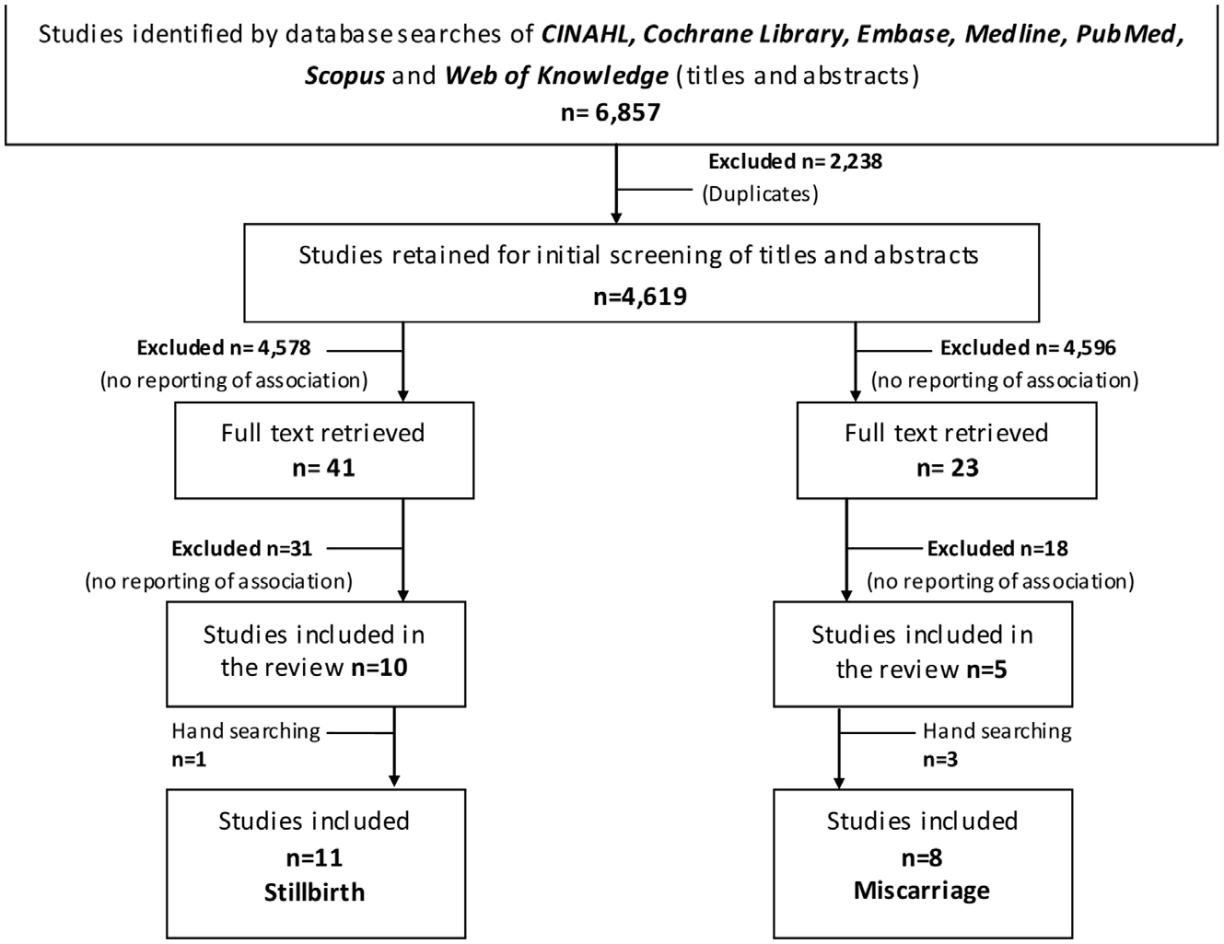
Selection of studies for inclusion in the systematic review.

We retrieved 4,619 non-duplicated studies (Figure 1), of which 41 included data for the association between mode of delivery and stillbirth and 23 included data for the association between mode of delivery and spontaneous miscarriage. The full text for these articles was reviewed for eligibility. The most frequent reason for study exclusion was absence of the outcome or the exposure of interest or study designs other than those cited in the inclusion criteria, followed by letters, reviews or editorials. For the stillbirth review, two studies [68], [69] were excluded to avoid duplication as the data used in each came from the same Scottish database as a later study [70], which is included in the final review. Ten studies were eligible for inclusion, and one additional study was identified from cross-checking the reference lists, yielding a total of eleven studies for inclusion in the stillbirth review, nine cohort studies [53], [70]–[77] and two cross-sectional studies [78], [79]. For the miscarriage review, five studies met the inclusion criteria, and three additional studies were identified from cross-checking of the reference lists, yielding a total of eight articles for inclusion in the review; seven cohort studies [51], [52], [80]–[84] and one case-control study [85]. Hemminki et al. [83] reported on two separate cohorts, and consequently two different risk estimates representing each cohort are presented.

### Characteristics of Studies Included in the Stillbirth Review

A summary of the study characteristics can be found in Table 1. The definition of stillbirth used varied by study with different cut-offs for gestational age and birth weight. One third of the studies defined stillbirth from an early gestational age (> = 20 weeks) [71], [73], [78] or (> = 22 weeks) whilst two thirds used a later gestation (> = 23 weeks) [53], [76], (> = 24 weeks) [70], [74], [77] and (> = 28 weeks) [75]. Minimum birth weight defined as 400 g [71], [78], 500 g [72] or 1000 g [79] was used by four studies. Six studies included antepartum stillbirths [53], [70], [72], [74], [76], [77]. All but one study [75] excluded women with multiple births (twins or greater) and four studies excluded deaths of fetuses as a result of congenital anomalies [53], [70], [73], [75]. Ten of the included studies were conducted in high-income countries including: the UK [70], [75], Germany [53], [74], Australia [71], [78], Israel [72], the USA [73], [76] and Canada [77] and one study was conducted in a low-income country, Nigeria [79]. Ascertainment of stillbirth was confirmed in the studies through one or more of the following methods: hospital database(s) [53], [70]–[75], [77], [78], patient charts [72], [74], [79] or nationwide registers [70]. Four studies used the WHO International Classification of Disease (ICD) codes [70], [75], [76], [79]. The causes of stillbirth were identified in two studies [70], [77] using Wigglesworth’s classification system [86], [87]. Another study [71] used more recent criteria by Whitfield [88] to classify stillbirth and two studies used autopsy [70], [77].

**Table 1:** Characteristics of studies included in the stillbirth review.

Cohortstudies	Country/Studyperiod	Study design and data source	Cohort size	Number of stillbirths in cohort	Parity	Stillbirth definition^#^	Exclusions
Gray et al^75^(2007)	UK,1968–1989	Retrospective population-based cohort using the Oxford Record Linkage Study (ORLS) databaselinked birth/death registries	81,784	467	Multiparous	>28 weeks; included *explained* and *unexplained* stillbirths	Deliveries <28 weeks and >43 weeks gestation, congenital anomalies, negative/implausible inter-pregnancy intervals, any missing data
Franz et al^53^(2009)	Germany,1987–2005	Retrospective population-based cohort using perinatal survey data from the Bavaria region database(98% complete)	629,815	1,386	Primiparous	>23 weeks; included *unexplained antepartum* stillbirths	Multiple births; congenital anomalies, restricted to maternal age within 11–54 years and gestational age within 23–42 completed weeks
*Kennare et al^71^(2007)	Australia,1998–2003	Retrospective population-based cohort using the South Australian Perinatal database	36,038	183	Primiparous	>20 weeks; 400 g; included *unexplained* stillbirths and *unspecified* stillbirths. No estimate for *explained* stillbirths provided	Late terminations of pregnancy, multiple births, intrapartum and postpartum death
Smith et al^70^(2007)	Scotland,1992–2001	Retrospective cohort usingnationwide linked pregnancy (SMR2)and national perinatal death registries	133,163	357	Primiparous	>24 weeks; included *unexplained antepartum* stillbirths	Missing data on gestational age, infant sex or birth weight, gestational age outside 24–43 weeks, congenital anomalies, history of stillbirth/neonatal death, multiple births
Wood et al^77^(2008)	Canada,1991–2004	Retrospective cohort using aCanadian Perinatal Health Programdatabase including 81 hospitals	158,502	639	Primiparous	>24 weeks; included *unexplained antepartum* stillbirths	Multiple births, missing data
Ohana et al^72^(2011)	Israel,1988–2009	Retrospective population -based cohort using Soroka UniversityMedical Center Perinatal database	228,293	7.4 per 1000	Multiparous	>22 weeks; 500 g; included antepartum *unspecified* stillbirths	Intrapartum and postpartum deaths, multiple pregnancies
Reddy et al^76^(2010)	USA,2002–2008	Retrospective cohort of women enrolled in the Consortium on Safe Labor study including 12 clinical centres & 19 hospitals	174,809	5.2 per 1000	Multiparous	>23 weeks; included antepartum *unspecified* stillbirths	Multiple gestations, intrapartum stillbirths, neonatal deaths, missing data
Richter et al^74^(2007)	Germany,1993–1999	Retrospective population-based cohort using Berlin PerinatalRegistry database	62,698	231	Primiparous	>24 weeks; included antepartum *unspecified* stillbirths	Multiple pregnancies, deliveries before 24 weeks gestation
Salihu et al^73^(2006)	USA,1978–1997	Retrospective population-based cohort using Missouri maternally linked data	396,441	1,612	Primiparous	>20 weeks; includedu*nspecified* stillbirths	Restricted to gestation >20 weeks and <44 weeks, excluded congenital anomalies, multiple births
Olusanya et al^79^(2009)	Nigeria,2005–2007	Unmatched case-control cross-sectional study using a singlehospital register of births in Lagos	7,216	146.7 per 1000	Multiparous	>28 weeks; 1000 g; included *unspecified intrapartum* term stillbirths	Multiple pregnancies, missing data on gestational age and weights
Cross sectional studies	Country/Studyperiod	Study design and data source	Cohort size	Number of stillbirthsin cohort	Parity	Stillbirth definition*	Exclusions
Taylor et al^78^(2005)	Australia,1998–2002	Cross-sectional study using linked databases including the Midwives Data Collection [MDC] and the Inpatients Statistics Collection [ISC]	136,101	582	Primiparous	>20 weeks; 400 g; included u*nspecified* stillbirths	Multiple births, missing data

**Figure 2:**
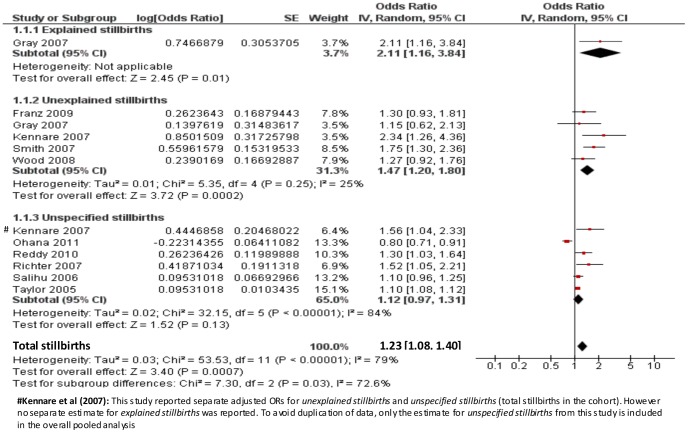
Random-effect model of the risk of subsequent stillbirth associated with Caesarean delivery compared with vaginal delivery from 10 published studies including 1,958,292 women and 6,920 events by cause of stillbirth (explained, unexplained, unspecified).

**Figure 3:**
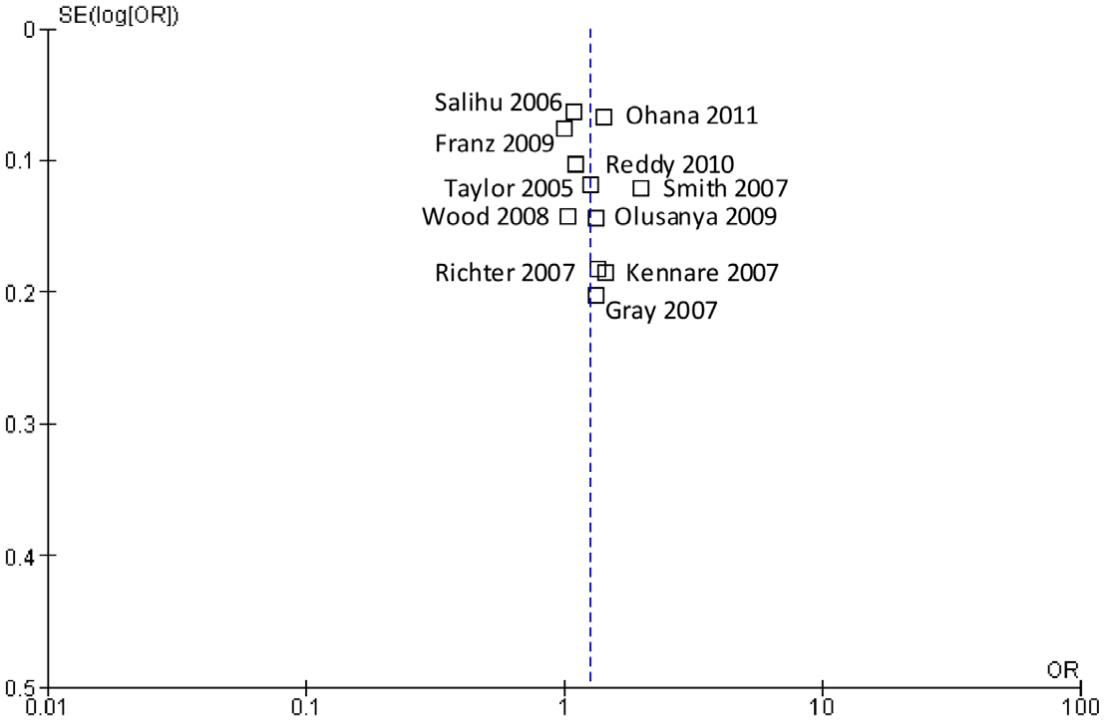
Funnel plot assessing publication bias in the risk of subsequent stillbirth associated with Caesarean delivery compared with vaginal delivery from eleven published studies.

For the primary analysis, data were available on 1,961,829 pregnancies and 7,308 (0.37%) events. No matched groups were used in any of the eleven studies. Only one study did not adjust for confounders and is not included in the meta-analysis [79]. A crude OR of 1.08 (95% CI 0.77, 1.52) for risk of subsequent stillbirth among women with a prior Caesarean section was reported. Most studies adjusted for the following potential confounders: maternal age, smoking, history of pregnancy loss, gestational age and parity. Adjustment for other potential confounders including BMI, socioeconomic status, marital status, maternal height, birth weight, medical complications such as diabetes or hypertension and race/ethnicity varied between the studies. Three studies reported a sample size or power calculation [71], [75], [77]. A random-effects model is reported due to considerable heterogeneity between the studies in the fixed-effect model (I^2^ = 84.2%, P = >0.00001). The pooled adjusted OR of stillbirth among women with previous Caesarean delivery versus vaginal delivery was 1.23 (95% CI 1.08, 1.40) (Figure 2). Inspection of the funnel plot (Figure 3) did not indicate evidence of publication bias.

### Subgroup Analyses

**Figure 4:**
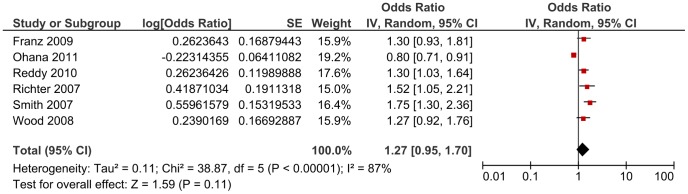
Sub-group analyses using a random-effect model of the risk of subsequent stillbirth associated with Caesarean delivery compared with vaginal delivery by cause of stillbirth (antepartum stillbirths only).

Subgroup analysis by cause of stillbirth yielded an OR of 1.47 (95% CI 1.20, 1.80) for studies including unexplained stillbirths, an OR of 2.11 (95% CI 1.16, 3.84) for the single study which reported an estimate for explained stillbirths and an OR of 1.12 (95%CI 0.97, 1.31) for those studies which included unspecified stillbirths (i.e. did not state whether the stillbirths were explained or unexplained and/or antepartum or intrapartum) (Figure 2). The Chi^2^ estimate to test for subgroups differences was 7.30 (P = 0.03). When studies which reported including antepartum stillbirths only were separately analysed, an OR of 1.27 (95% CI 0.95, 1.70, Chi^2^ 38.87) was generated (Figure 4).

### Sensitivity Analyses

**Figure 5:**
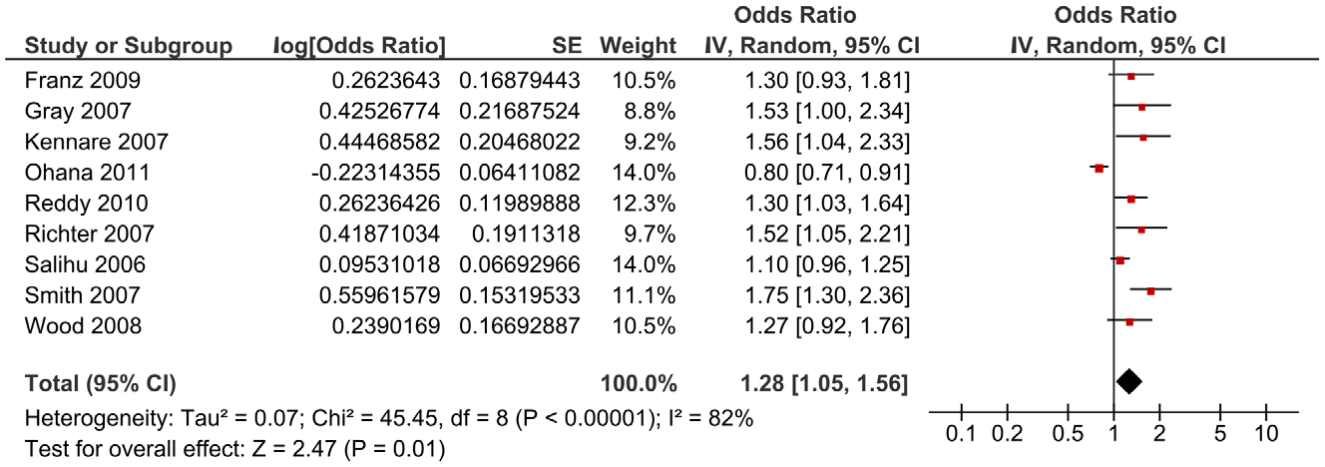
Sensitivity analyses using a random-effect model of the risk of subsequent stillbirth associated with Caesarean delivery compared with vaginal delivery by study design (cohort versus cross-sectional).

**Figure 6:**
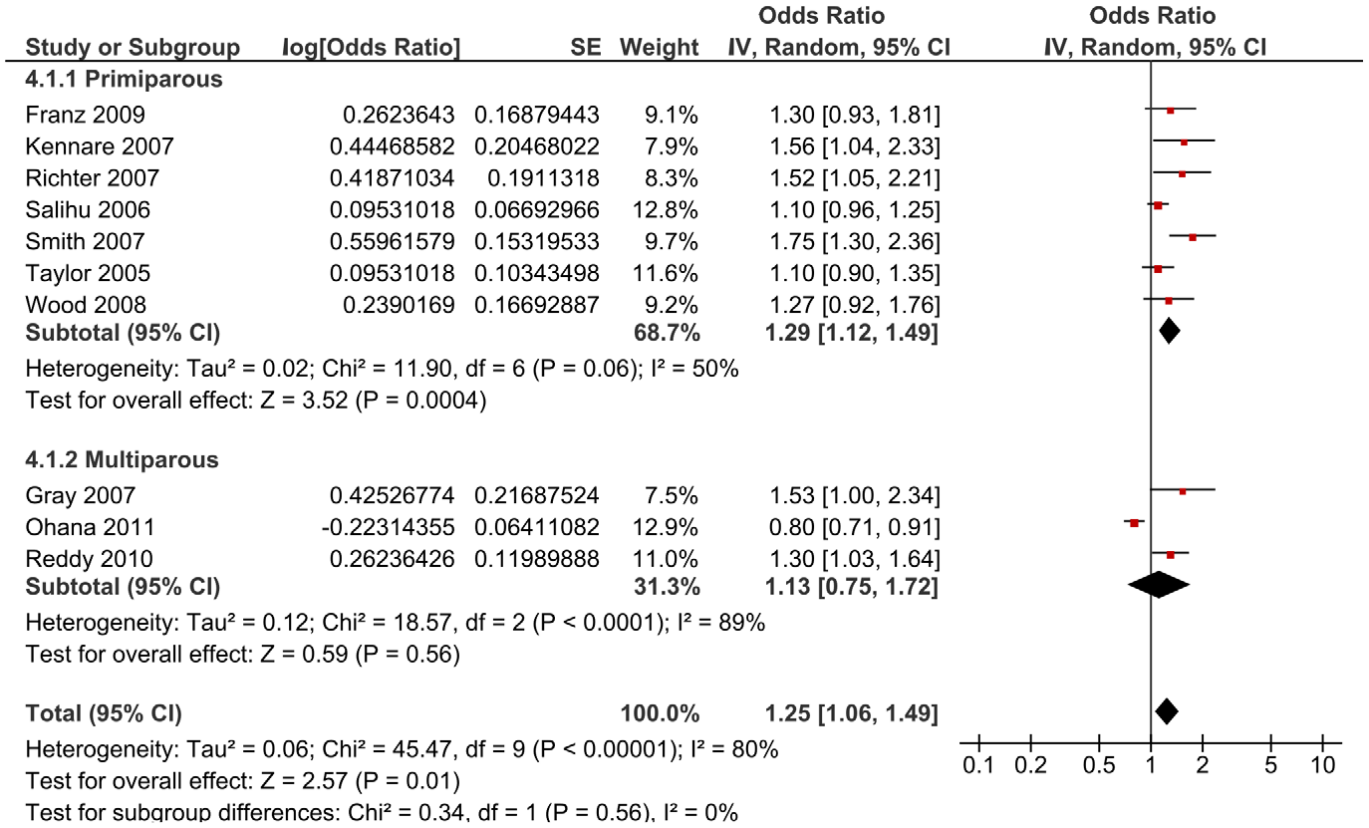
Sensitivity analyses using a random-effect model of the risk of subsequent stillbirth associated with Caesarean delivery compared with vaginal delivery parity (primiparous women versus multiparous women).

When the single cross-sectional study [78] was omitted, there was a small shift in the OR [from 1.23, 95% CI 1.08, 1.40 to 1.28, 95% CI 1.05, 1.56] (Figure 5). Separate analyses by parity produced an OR of 1.29 (95% CI 1.12, 1.49, Chi^2^ 11.90) for primiparous women, and an OR estimate of 1.13 (95% CI 0.75, 1.72, Chi^2^ 18.57) for multiparous women (Figure 6).

The PAR for all stillbirths in the total population attributed to previous Caesarean section was calculated to be 0.03551 per 100 and the PAR% estimated at 3.6%. PAR for unexplained stillbirths was 0.067 per 100 with a PAR% of 6.7% (data not shown).

### Characteristics of Studies Included in the Miscarriage Review

A summary of the study characteristics can be found in Table 2. The definition of miscarriage used varied by study and information provided was limited in terms of the gestational age cut-off and minimum birth weight. Two studies defined miscarriage using ICD codes [51], [83]. Miscarriage was simply defined as ‘spontaneous abortion’ or ‘miscarriage’ by the remaining studies. Five studies excluded women with multiple births (twins or greater) [80]–[84]. All of the included studies were conducted in high-income countries including: the USA [81], [85], Finland [51], Scotland [80], [82], [84], England [52] and Sweden [83]. Miscarriage diagnosis was confirmed in the studies through one or more of the following methods: hospital database(s) [52], [82], [84], patient charts [85], interviewing of women [85], survey data [81] or nationwide registers [51], [80], [83]. Two studies cited using the WHO ICD codes [51], [83]. Two studies [52], [80] divided the Caesarean group by indication (emergency or elective). For the other studies, it was assumed that the exposed group included all Caesareans. No study distinguished between early (6±12 weeks gestation) or late miscarriage (12±24 weeks gestation). Five studies used frequency matching by one or both of the following: age [51], [52], [81], [83], [85] and date of delivery [51], [52], [81]. Adjustment for potential confounders (marital status, deprivation, birth weight percentile, infant sex, maternal age, maternal height and method of induction) was only performed in one study [80] in the miscarriage review. None of the studies reported a sample size or power calculation. For the primary analysis of miscarriage, data were available on 147,017 women, of which 12,682 (8.6%) were reported to have experienced a miscarriage. Results of each study included in the miscarriage review are presented separately (Table 3) due to significant heterogeneity as a result of lack of adjustment for confounding. Two studies [51], [82] reported a statistically significant increase in odds of miscarriage following Caesarean delivery by 32% and 22% respectively. However, such results must be interpreted with caution as adjustment for confounders was not possible. Only one study [52] reported a reduction in the odds of miscarriage following Caesarean delivery, however, this was not statistically significant.

**Table 2:** Characteristics of studies included in the miscarriage review.

Study (year)	Region/Study period	Study design anddata source	Totalpopulation	Number ofmiscarriages in thecohort	Miscarriagedefinition	Exclusions
LaSalaet al^85^ (1987)	New York, USA; 1978	Retrospective case-control studyusing age and parity matched controls retrieved from the daily obstetric logbook records in hospital	570	23	Not definedother than ‘spontaneous abortion’	Missing data; women who were sterilised during the same hospitalisation
Hemminkiet al^51^ (1996)	Finland; 1987–1993	Retrospective cohort using linked nationwide birth & hospital registers	16,473	1,565	ICD-9 codes(631, 632, 634)	Implausibly short inter-pregnancy intervals
Mollisonet al^84^ (2005)	Aberdeen, Scotland;1980–1997	Prospective population-based cohort using data from the AberdeenMaternity Hospitaldatabank	25,371	1,475	‘Early fetal demise’ = ‘spontaneous or missed miscarriage’	Multiple births; stillbirths
Tower et al^52^ (2000)	Nottingham, UK; 1992–1993	Prospective cohort using data from a single hospital maternityinformation system	1,152	113	Not definedother than ‘miscarriage’	None stated
Hemminki^83^ (1986)	Sweden; two cohortsfollowed in 1973and 1976	Retrospective cohort using multiple nationwide hospital dischargeregistries forming the Swedish Birth Registry	1973 = 5,184; 1976 = 7,734	558	ICD-8 code(643)	Women with a hysterectomy; nationalities other than Swedish; multiple births; congenital anomalies; birth weight <2000 g; neonatal deaths;
Hemminkiet al^81^ (1985)	USA; three cohortsfollowed in 1973,1976 and1983	Retrospective cohort using cross-sectional data of women includedin the National Survey of Family Growth (NSFG), excluding Alaska and Hawaii, conducted by the National Centerfor Health Statistics (NCHS)	812	94	Not definedother than ‘spontaneous abortion’	Women outside of 15–44 years of age; multiple deliveries; history of recurrent miscarriage; history of stillbirth; missing data; infants weighing less than 1500 g at birth; infants dying within one year of birth
Hall et al^89^ (1989)	Aberdeen, Scotland; 1964–1983	Prospective cohort using data fromthe Aberdeen Maternity and Neonatal Databank	22,948	1,072	Not definedother than ‘miscarriage’	Multiple births; stillbirths
Smith et al^80^ (2006)	Scotland;1980–1984	Retrospective population-basedcohort study using the Scottish Morbidity records (SMR2) database of allmaternity hospitals	109,991	8,036	Not definedother than ‘spontaneous early pregnancy loss’	Multiple births; preterm births; Perinatal deaths; births outside the range 37–43 weeks gestation; missing values;

**Abbreviations: ICD,** International Classification of Diseases.

**Table 3:** Individual study estimates of Caesarean delivery and risk of subsequent miscarriage.

Study	Sub-category	Crude OR	95% CI
Hall et al^80^ (1989)		1.32	1.06–1.65
Hemminki et al^79^ (1985)		1.10	0.72–1.69
Hemminki^81^ (1986)	Cohort 1∶1973	1.10	0.82–1.47
	Cohort 2∶1976	1.12	0.90–1.38
Hemminki et al^51^ (1996)		1.22	1.10–1.36
LaSala et al^83^ (1987)		1.26	0.54–2.92
Mollison et al^82^ (2005)		1.06	0.92–1.23
Smith et al^78^ (2006)		1.07	1.00–1.15
Tower et al^52^ (2000)		0.76	0.48–1.18

### Quality Assessment

Quality assessment of the included studies (Tables 4, 5) was based on a bias classification tool estimating six types of bias (Appendix S2). Overall, the risk of bias for the studies included in the stillbirth review was considered ‘minimal’ and ‘moderate’ for the miscarriage studies.

**Table 4:** Quality assessment of studies included in the stillbirth review.

Study	Selection bias	Exposure bias	Outcome assessment bias	Confounding factor bias*	Analytical bias	Attrition bias	Overall likelihoodof bias
Franz et al^53^(2009)	Minimal (populationbased data registry with 98% coverage)	Low (recorded in dataset butmay be under-reportedbefore 1997)	Low (recorded in dataset)	Minimal (adjusted for diabetes, smoking, advanced maternal age, previouspremature birth, small-for-gestational age infant, neonatal death and stillbirth)	Minimal (Cox regression andKaplan Meier curves withadjustment for confounders)	Minimal (all subjects from initiation tofinal outcomeaccounted for)	Minimal
Gray et al^73^(2007)	Minimal (linked statistical dataset of over 80,000 women)	Low (recorded in linked dataset which used birth registrations, death certs and hospitalinformation systems)	Low (recorded in linked database, ICD-8 andICD-9 codes used)	Minimal (adjusted for maternal age,parity, social class, history of pregnancyloss, BMI and smoking)	Minimal (Cox regression with adjustment used)	Minimal (all subjects accounted for)	Minimal
Kennare et al^69^(2007)	Minimal (perinatal database-97% agreement with hospital records)	Low (recorded in dataset)	Low (recorded indatabase and used classification system)	Minimal (adjusted for hypertension, gestation, smoking, age, race, history of pregnancy loss)	Minimal (multiplelogistic regression with adjustment forconfounders)	Minimal (no loss to follow up)	Minimal
Ohana et al^70^(2011)	Minimal (non-selective population-based data)	Low (perinatal database with information recordedimmediately after delivery)	Low (perinatal database with informationrecorded immediatelyafter delivery)	Minimal (adjusted for history of pregnancy loss, hypertension, maternal age,gestational diabetes, ethnicity and maternal complications)	Minimal (multiple logistic regression with adjustment for key confounders)	Minimal (no loss to follow up)	Minimal
Olusanya et al^77^(2009)	Low (one hospital in Nigeria, select race)	Minimal (hospital records used)	Minimal (hospital records, specific definition used)	Moderate (no adjustment for confounders, no matching)	Moderate (no sample size calculation, no adjustment for confounders,	Minimal (all patients accounted for)	Moderate
Reddy et al^74^ (2010)	Minimal (multiplehospitals in USA included,large sample)	Low (hospital database used)	Low (hospitaldatabase used,specific definition)	Minimal (adjusted for race, maternal age, marital status, health insurance, parity, preterm birth, diabetes, hypertension, smoking, alcohol use, BMI and HIV/AIDS status)	Minimal (univariate andmultivariate Cox regressionanalysis performed)	Minimal (all data accounted for)	Minimal
Richter et al^72^(2007)	Minimal (all secondbirths in Berlin)	Low (Berlin database)	Low (hospitaldatabases used)	Minimal (Maternal age, weight, nationality and obstetric, medical and antenatalhistory and smoking during pregnancy)	Minimal (univariate andmultivariate logistic and Cox regressionanalysis)	Minimal (all data inclusions andexclusions detailed)	Minimal
Salihu et al^71^(2006)	Minimal (linked cohort data files from 1978–97in Missouri- validated)	Minimal (validated vital records database)	Minimal (validated database used)	Minimal (adjusted for maternal age, parity, marital status, educational status, smoking, BMI, history of small-for-gestational age or preterm infant)	Minimal (multivariate logistic regression with adjustment)	Minimal (no loss to follow up)	Minimal
Smith et al^68^(2007)	Minimal (all births in Scotland, quality assured database)	Minimal (validated databasewith 99% coverage)	Minimal (validated database with ICD-9codes used)	Minimal (adjusted for socioeconomic deprivation, smoking, maternal age and height and marital status)	Minimal (Survival analysis using Kaplan Meier and Coxproportional hazards models)	Minimal (all subjects accounted for)	Minimal
Taylor et al^76^(2005)	Minimal (all births inNew South Wales)	Low (Linked populationdatabases)	Low (database used)	Minimal (adjusted for maternal age,smoking, health insurance status, ethnicity, SES, diabetes, hypertension, gestationalage and history of stillbirth)	Minimal (Univariate andmultivariate logistic regression used)	Minimal (all subjects accounts for)	Minimal
Wood et al^75^(2008)	Minimal (all birthsbetween 1991–2004 in Alberta)	Minimal (validated perinatal database including 81+ hospitals	Minimal (validated database)	Minimal (adjusted for maternal age,diabetes, hypertension, smoking and BMI)	Minimal (multivariate logistic regression)	Minimal (no loss to follow up)	Minimal

**Table 5:** Quality assessment of studies included in the miscarriage review.

Study	Selection bias	Exposure bias	Outcome assessment bias	Confounding factor bias*	Analytical bias	Attrition bias	Overall likelihoodof bias
LaSala et al^83^(1987)	Minimal (all womengiving birth in the New York Hospital in 1978)	Minimal (recorded from hospital chart)	Minimal (assessment from hospital records)	Moderate (no adjustmentfor confounding stated)	Moderate (analyses not accounting for common statistical adjustment, no sample size calculation reported)	Moderate (>20% attrition but reasons for loss to follow-up explained)	Moderate
Hemminki et al^51^ (1996)	Minimal (validated nationwide registerswith 97% coverage)	Low (recorded from nationwide register using ICD-9 codes)	Low (nationwide register used with ICD-9 codes)	Moderate (adjustmentfor confounding not reported)	Minimal (matched sample used, no sample size calculation reported)	Minimal (no loss to follow-up)	Moderate
Mollison et al^82^ (2005)	Minimal (select groupbut eligibility explained)	Low (assessment from dataset)	Low (assessment from dataset)	Moderate (no adjustmentfor confounding)	Minimal (sample matched,no sample size calculation)	Minimal (no loss to follow-up)	Moderate
Tower et al^52^(2000)	Minimal (select groupbut eligibility explained)	Low (assessment from dataset)	Low (assessment from dataset)	Moderate (no adjustmentfor confounders)	Minimal (sample matched,no sample size calculation)	Minimal (no loss tofollow-up)	Moderate
Hemminki^81^(1986)	Minimal (largepopulation-baseddataset used)	Low (Swedish birth and hospital registries used)	Low (recorded fromnationwide dataset)	Moderate (no adjustmentfor confounders)	Low (only t-test used)	Minimal (no loss to follow-up)	Moderate
Hemminki et al^79^(1985)	Minimal (nationwide survey)	Minimal (personal interview with mothers)	Minimal (personal interviewwith mothers)	Moderate (no adjustmentfor confounders)	Low (chi-square test and Kaplan Meier curves)	Low (no loss to follow-up)	Moderate
Hall et al^80^(1989)	Minimal (stable homogenous population giving birth between1964 and 1983)	Minimal (Aberdeen Data Bankused)	Minimal (Large database used)	Moderate (no adjustmentfor confounders)	Moderate (no statistical details cited)	Low (none stated)	Moderate
Smith et al^78^(2006)	Minimal (109,000women giving birth between 1980–1999 in Scotland)	Minimal (validated Scottish Morbidity Record database)	Minimal (Validated Scottish Morbidity Record database)	Minimal (adjusted for marital status, deprivation, birth weight, infant sex, maternal age, maternal height)	Minimal (linear regression, logistic regression and X^2^ tests used)	Minimal (No loss to follow-up)	Minimal

* Assessment of confounding factor bias was done by evaluation of each study’s assessment of potential confounders by four methods: adjustment with regression, matching, assessment of potential confounders on univariate analyses that were found not to be significantly different between groups, and assessment of potential confounders on univariate analyses that were different between groups and not controlled for.

NA = Not applicable.

### Heterogeneity Assessment

#### Stillbirth

The characteristics of the included studies are shown in Table 1. All ten studies included in the stillbirth meta-analysis were from high income countries. In addition all sampled retrospective population-based cohorts using hospital or register-based databases. Variations in the definition of stillbirth used may account for some of the heterogeneity observed. Definitions ranged from greater than 20 weeks to greater than 28 weeks and included all, explained or unexplained stillbirths. The I^2^ statistic was used to measure statistical heterogeneity and varied from 0% to 89%. Heterogeneity due to cause and timing of stillbirth used and by study design and parity may explain some or all of the observed heterogeneity.

#### Miscarriage

The characteristics of the included studies are shown in Table 2. All eight studies were from high income countries. Variations in the definition of miscarriage used may account for some of the heterogeneity observed as there was limited information available on gestation and birth weight. Furthermore, only one study adjusted for confounding.

## Discussion

The overall findings of the meta-analysis suggest that women with a previous Caesarean delivery have a 23% increased odds of subsequent stillbirth compared to women with a previous vaginal delivery. The significant effect of Caesarean delivery on stillbirth was present in the overall meta-analysis and persisted in the subgroup analysis by cause of stillbirth (explained, unexplained) as well as the sensitivity analyses by study design (cohort studies only) and parity (primiparous women only). A reduction of 0.036 stillbirths per 100 population (exposed and unexposed) is expected if women were not exposed to a Caesarean delivery (*PAR = *0.036 per 100). This represents a 3.6% reduction of the incidence in the population (*PAR*% = 3.6%). For unexplained stillbirths only, such a reduction represents a 6.7% decrease in the incidence in the population (*PAR*% = 6.7%). The results for subsequent risk of miscarriage are less pronounced and due to lack of adjustment for confounders no meta-analysis was conducted. The single study in the miscarriage analysis that included adjustment for confounders did not report a significant association. This would suggest that confounding may explain some or all of this increased risk. Overall the results of this systematic review and meta-analysis underscore the importance of further research into the association between mode of delivery and risk of subsequent miscarriage or stillbirth.

To date, there are major gaps in the understanding of the aetiology of stillbirth and miscarriage. Prior to this review, the association between previous Caesarean delivery and stillbirth or miscarriage in subsequent pregnancies was unclear. Flenady et al. [1] undertook a meta-analysis to investigate potential risk factors for stillbirth in high income countries and also found an increased odds of stillbirth following Caesarean delivery, with unexplained stillbirths attributable to an even greater increase in odds, similar to the findings of this systematic review and meta-analysis. Given the increasing use of Caesarean delivery for reasons including maternal choice, increased maternal age, fear of litigation among clinicians and repeat Caesareans, even a small increase in risk would have important implications at a population level. Despite surgical advances, Caesarean delivery continues to be associated with a significantly increased risk of maternal morbidity including haemorrhage, chronic pain, pelvic adhesion, sub fertility, placenta accrete [89] and death compared with vaginal delivery [90], [91], as well as an increased risk of perinatal morbidity [92] in subsequent deliveries. Nevertheless, it is also important to comment on the potential benefits associated with Caesarean deliveries including reduced urinary incontinence [93] and its necessity in emergency situations such as breech presentation [94], prematurity [95] and dystocia [96]. Caesarean delivery today is a much safer operation due to advances in anaesthesia, antibiotics, surgical training and blood transfusion [97]. However, some of these indications including breech presentation for Caesarean delivery in a previous pregnancy may confer an increased risk of stillbirth or miscarriage in the next pregnancy. Therefore confounding by indication in the previous pregnancy may explain the increased risk generated in the overall pooled analysis. However the persistence of the association between mode of delivery and stillbirth in sensitivity analyses including by parity suggests that the association may be real. Moreover, with such an exponential rise in Caesarean delivery, any potential association between mode of delivery and adverse pregnancy outcomes such as stillbirth and miscarriage is of major public health and health policy importance in the future.

### Strengths and Limitations of the Review

A major strength of the review includes the comprehensive literature search which included seven databases using a wide-ranging collection of search terms and two reviewers. The heterogeneity between included studies was examined using appropriate sensitivity analyses and overall pooled estimates were generated to quantify the effect of Caesarean delivery on subsequent stillbirth and miscarriage. However, several limitations were found in some of the included observational studies. Data deficits or data of poor and variable quality existed with varying definitions of both outcomes used. Only four studies for stillbirth [70], [75], [76], [79] and two for miscarriage [51], [83] used of the International Classification of Disease (ICD) coding. Two studies referred to Wigglesworth’s criteria for stillbirth [70], [77] and one study used Whitfield’s criteria [71], both of which have their own strengths and weaknesses. As previously discussed, there was a wide variation in the definition of stillbirth as well as miscarriage used in the studies included in this review. This is to a degree due to varying definitions of stillbirth and miscarriage in different countries as well as the definitions changing over time. Such variation may as a result over or under estimate stillbirth risk, conditional on whether the association is dependent on gestational age or not. No study in the miscarriage review included a definition of miscarriage according to gestation (commonly <20–22 weeks), and only two studies used ICD codes [51], [83]. However, nationwide registry data was used by one large study in the stillbirth review [70] and three in the miscarriage review [51], [80], [83], with the majority having almost complete coverage, and examination of data recording quality. The other studies used patient records, interviews, survey data, and autopsy, which may be subject to errors in the form of miscoding or information bias. Small or inadequate sample sizes in the case-control studies with the largest effect estimates based on small sample sizes, as well as heterogeneous entry criteria may also limit the findings. Only three studies reported a power or sample size calculation in the stillbirth analysis and none were cited in the miscarriage analysis. However, the majority of included studies in both reviews were cohort studies with adequate power.

While adjustment for potential confounders varied across the studies, ten out of the 11 studies included in the stillbirth review adjusted for the main confounders. However, only one study adjusted for confounders in the miscarriage review and no meta-analysis was performed as a result. The quality of the outcome data in the retrospective studies may also be subject to bias in the form of under reporting of the events in the hospital records, recall bias and measurement bias. Selection of an appropriate, unbiased comparison group in case-control studies may also bias results in any direction.

### Conclusions and Implications

The overall findings would suggest that women who previously delivered by Caesarean have a 23% increased risk of subsequent stillbirth compared to women who have previously delivered vaginally. However, studies included in the miscarriage review were of poor methodological quality and overall the results are inconsistent. The results must therefore be interpreted with caution. There are a multitude of medical, social and personal factors affecting decisions regarding mode of delivery. Caesarean delivery should be performed when medically required. However, the risk of subsequent adverse pregnancy outcomes, such as stillbirth and miscarriage, should not only influence medical decision-making, but also enter the patient and clinician discourse. These results are timely given the recent revision of the NICE guidelines, which for women requesting a Caesarean section, if after discussion and offer of support (including perinatal mental health support for women with anxiety about childbirth), a vaginal birth is still not acceptable, a Caesarean section is offered.

Risks and benefits to not only the current birth but also future pregnancies and births should be carefully considered. This particularly concerns primiparous women, who are likely to want more children. Research in the future should preferably use population-based data with lengthy follow-up. Clearly defined exposure and outcome criteria should be central to the research and information on potential key confounders would be vital for investigating any risks related to Caesarean delivery. A universal definition across all countries for stillbirth and miscarriage is recommended, for the recording of vital statistics and for research to be more comparative and of higher impact and better methodological quality.

## Supporting Information

Appendix S1 **Search terms used to search CINAHL, the Cochrane Library, Embase, MEDLINE, PubMed, Scopus and Web of Knowledge to identify studies on the association between Caesarean section and risk of stillbirth or spontaneous miscarriage.**
(DOC)

Appendix S2 **Bias classification tool for study quality assessment.**
(DOC)
