# Correction: Caesarean Delivery and Subsequent Stillbirth or Miscarriage: Systematic Review and Meta-Analysis

**DOI:** 10.1371/journal.pone.0110018

**Published:** 2014-09-30

**Authors:** The PLOS ONE Staff

There is an error in the last sentence of the Data synthesis section of the Abstract. The correct sentence is: No study reported adjusted estimates in the miscarriage review, therefore results are presented individually.

There is an error in the 13th sentence of the Characteristics of Studies Included in the Miscarriage Review section of the Results. The correct sentence is: No study in the miscarriage review reported adjusting for potential confounders.

There is an error in the last sentence of the Miscarriage subsection of the Heterogeneity Assessment in the Results. The correct sentence: Furthermore, no study adjusted for confounding.

The third to last sentence in the first paragraph of the discussion should be removed.

There is an error in the second sentence of the second paragraph in the Strengths and Limitations of the Review section of the Discussion. The correct sentence is: No study adjusted for confounders in the miscarriage review and so a meta-analysis was not performed as a result.

There are errors in Table 5. The Confounding Factor Bias and Analytical Bias columns for Smith et al (2006) are incorrect. Please see the correct table here:

**Table 5:** Quality assessment of studies included in the miscarriage review.

Study	Selection bias	Exposure bias	Outcome assessment bias	Confounding factor bias*	Analytical bias	Attrition bias	Overall likelihood of bias
LaSala et al^83^ (1987)	Minimal (all women giving birth in the New York Hospital in 1978)	Minimal (recorded from hospital chart)	Minimal (assessment from hospital records)	Moderate (no adjustment for confounding stated)	Moderate (analyses not accounting for common statistical adjustment, no sample size calculation reported)	Moderate (>20% attrition but reasons for loss to follow-up explained)	Moderate
Hemminki et al^51^ (1996)	Minimal (validated nationwide registers with 97% coverage)	Low (recorded from nationwide register using ICD-9 codes)	Low (nationwide register used with ICD-9 codes)	Moderate (adjustment for confounding not reported)	Minimal (matched sample used, no sample size calculation reported)	Minimal (no loss to follow-up)	Moderate
Mollison et al^82^ (2005)	Minimal (select group but eligibility explained)	Low (assessment from dataset)	Low (assessment from dataset)	Moderate (no adjustment for confounding)	Minimal (sample matched, no sample size calculation)	Minimal (no loss to follow-up)	Moderate
Tower et al^52^ (2000)	Minimal (select group but eligibility explained)	Low (assessment from dataset)	Low (assessment from dataset)	Moderate (no adjustment for confounders)	Minimal (sample matched, no sample size calculation)	Minimal (no loss to follow-up)	Moderate
Hemminki^81^ (1986)	Minimal (large population-based dataset used)	Low (Swedish birth and hospital registries used)	Low (recorded from nationwide dataset)	Moderate (no adjustment for confounders)	Low (only t-test used)	Minimal (no loss to follow-up)	Moderate
Hemminki et al^79^ (1985)	Minimal (nationwide survey)	Minimal (personal interview with mothers)	Minimal (personal interview with mothers)	Moderate (no adjustment for confounders)	Low (chi-square test and Kaplan Meier curves)	Low (no loss to follow-up)	Moderate
Hall et al^80^ (1989)	Minimal (stable homogenous population giving birth between 1964 and 1983)	Minimal (Aberdeen Data Bank used)	Minimal (Large database used)	Moderate (no adjustment for confounders)	Moderate (no statistical details cited)	Low (none stated)	Moderate
Smith et al^78^ (2006)	Minimal (109,000 women giving birth between 1980-1999 in Scotland)	Minimal (validated Scottish Morbidity Record database)	Minimal (Validated Scottish Morbidity Record database)	Moderate (no adjustment for confounders)	Low (Chi square tests)	Minimal (No loss to follow-up)	Minimal

*Assessment of confounding factor bias was done by evaluation of each study’s assessment of potential confounders by four methods: adjustment with regression, matching, assessment of potential confounders on univariate analyses that were found not to be significantly different between groups, and assessment of potential confounders on univariate analyses that were different between groups and not controlled for NA = Not applicable.
