# Supplementary material for: Caesarean Delivery and Subsequent Stillbirth or Miscarriage: Systematic Review and Meta-Analysis
Source: PLoS One. 2013 Jan 23;8(1):e54588. doi: 10.1371/journal.pone.0054588 (PMC3553078; doi:10.1371/journal.pone.0054588)
Supplement: Appendix S1 — Search terms used to search CINAHL, the Cochrane Library, Embase, MEDLINE, PubMed, Scopus and Web of Knowledge to identify studies on the association between Caesarean section and risk of stillbirth or spontaneous miscarriage. (DOC) [file pone.0054588.s001.doc]

| **Search terms** | **No of records returned** |
| --- | --- |
| ***For CINAHL (1981- November 11^th^ 2011 )LIMIT: humans*** |  |
| 1. Caesarean section | 1138 |
| 1. Caesarean sections | 246 |
| 1. Cesarean section | 3222 |
| 1. Cesarean sections | 379 |
| 1. Delivery, abdominal | 33 |
| 1. Deliveries, abdominal | 33 |
| 1. Abdominal delivery | 6 |
| 1. Abdominal deliveries | 6 |
| 1. C-section | 291 |
| 1. C-sections | 56 |
| 1. C section | 861 |
| 1. C sections | 220 |
| 1. Postcesarean section | 6 |
| 1. Postcaesarean section | 1 |
| 1. Caesarean delivery | 429 |
| 1. Cesarean delivery | 1607 |
| 1. Delivery, Caesarean | 429 |
| 1. Delivery, Cesarean | 1607 |
| 1. Mode of delivery | 1189 |
| 1. [#1 or #2 or #3 or #4 or #5 or #6 or #7 or #8 or #9 or #10 or #11 or #12 or #13 or #14 or #15 or #16 or #17 or #18 or #19] | 5879 |
| 1. Abortion, spontaneous | 2771 |
| 1. Spontaneous, abortion | 2771 |
| 1. Abortions, spontaneous | 591 |
| 1. Spontaneous, abortions | 591 |
| 1. Abortion, missed | 74 |
| 1. Missed, abortion | 74 |
| 1. Abortions, missed | 32 |
| 1. Missed, abortions | 32 |
| 1. Miscarriage | 3089 |
| 1. Miscarriages | 1170 |
| 1. Spontaneous, miscarriage | 149 |
| 1. Spontaneous, miscarriages | 42 |
| 1. Miscarriage, spontaneous | 149 |
| 1. Miscarriages, spontaneous | 42 |
| 1. **Incomplete miscarriage** | 29 |
| 1. **Miscarriage, incomplete** | 29 |
| 1. **Complete miscarriage** | 13 |
| 1. **Miscarriage, complete** | 13 |
| 1. **Inevitable miscarriage** | 12 |
| 1. **Miscarriage, inevitable** | 12 |
| 1. **Late miscarriage** | 78 |
| 1. **Miscarriage, late** | 78 |
| 1. **Early miscarriage** | 208 |
| 1. **Miscarriage, early** | 208 |
| 1. **Silent miscarriage** | 5 |
| 1. Abortion, incomplete | 164 |
| 1. Incomplete abortion | 164 |
| 1. Abortions, incomplete | 58 |
| 1. Incomplete, abortions | 58 |
| 1. Abortion, complete | 123 |
| 1. Complete, abortion | 123 |
| **Search terms** | **No. of records returned** |
| ***For PubMed (1966-November 11^th^ 2011 )LIMIT: human*** |  |
| 1. Abortions, complete | 37 |
| 1. Complete, abortions | 37 |
| 1. Abortion, inevitable | 30 |
| 1. Inevitable, abortion | 30 |
| 1. Abortions, inevitable | 4 |
| 1. Inevitable, abortions | 4 |
| 1. Abortion, tubal | 23 |
| 1. Abortions, tubal | 18 |
| 1. Tubal, abortion | 23 |
| 1. Tubal, abortions | 18 |
| 1. Recurrent, miscarriage | 186 |
| 1. Miscarriage, recurrent | 186 |
| 1. Recurrent, miscarriages | 64 |
| 1. Miscarriages, recurrent | 64 |
| 1. Septic, miscarriage | 5 |
| 1. Miscarriage, septic | 5 |
| 1. Anembryonic pregnancy | 12 |
| 1. **Fetal death in utero** | 30 |
| 1. **Intrauterine fetal death** | 155 |
| 1. **Embryonic demise** | 12 |
| 1. **Stillbirth** | 2207 |
| 1. [#21 or #22 or #23 or #24 or #25 or #26 or #27 or #28 or #29 or #30 or #31 or #32 or #33 or #34 or #35 or #36 or #37 or #38 or #39 or #40 or #41 or #42 or #43 or #44 or #45 or #46 or #47 or #48 or #49 or #50 or #51 or #52 or #53 or #54 or #55 or #56 or #57 or #58 or #59 or #60 or #61 or #62 or #63 or #64 or #65 or #66 or #67 or #68 or #69 or #70 or #71 or #72] | 7663 |
| 1. [#20 and #73] | 355 |
| 1. Article | 2540228 |
| 1. Cohort study | 30833 |
| 1. Case-control study | 12481 |
| 1. Systematic review | 47990 |
| 1. Review | 407331 |
| 1. Retrospective study | 22718 |
| 1. Prospective study | 51795 |
| 1. [#75 or #76 or #77 or #78 or #79 or #80 or #81] | 2543713 |
| 1. Longterm effects | 138 |
| 1. Long-term effects | 15282 |
| 1. Long-term complications | 3568 |
| 1. Longterm complications | 36 |
| 1. Pregnancy outcome | 3549 |
| 1. Postoperative complications | 20026 |
| 1. Subfertility | 378 |
| 1. Sub-fertility | 45 |
| 1. Subsequent fertility | 134 |
| 1. Secondary subfertility | 5 |
| 1. [#83 or #84 or #85 or #86 or #87 or #88 or #89 or #90 or #91 or #92] | 41677 |
| 1. **[#20 and #73 and #82 and #93]** | **123** |

| **Search terms** | **No of records returned** |
| --- | --- |
| ***For Medline (1966- Novemeber 11^th^ 2011)*** |  |
| 1. Caesarean section | 11173 |
| 1. Caesarean sections | 1516 |
| 1. Cesarean section | 38088 |
| 1. Cesarean sections | 3293 |
| 1. Delivery, abdominal | 3755 |
| 1. Deliveries, abdominal | 492 |
| 1. Abdominal delivery | 3755 |
| 1. Abdominal deliveries | 492 |
| 1. C-section | 465 |
| 1. C-sections | 145 |
| 1. C section | 26984 |
| 1. C sections | 14113 |
| 1. Postcesarean section | 245 |
| 1. Postcaesarean section | 16 |
| 1. Caesarean delivery | 5376 |
| 1. Cesarean delivery | 16751 |
| 1. Delivery, Caesarean | 5376 |
| 1. Delivery, Cesarean | 16751 |
| 1. Mode of delivery | 6485 |
| 1. [#1 or #2 or #3 or #4 or #5 or #6 or #7 or #8 or #9 or #10 or #11 or #12 or #13 or #14 or #15 or #16 or #17 or #18 or #19] | 91560 |
| 1. Abortion, spontaneous | 17478 |
| 1. Spontaneous, abortion | 17478 |
| 1. Abortions, spontaneous | 4770 |
| 1. Spontaneous, abortions | 4770 |
| 1. Abortion, missed | 1429 |
| 1. Missed, abortion | 1429 |
| 1. Abortions, missed | 281 |
| 1. Missed, abortions | 281 |
| 1. Miscarriage | 5064 |
| 1. Miscarriages | 2547 |
| 1. Spontaneous, miscarriage | 2139 |
| 1. Spontaneous, miscarriages | 1010 |
| 1. Miscarriage, spontaneous | 2139 |
| 1. Miscarriages, spontaneous | 1010 |
| 1. **Incomplete miscarriage** | 119 |
| 1. **Miscarriage, incomplete** | 119 |
| 1. **Complete miscarriage** | 196 |
| 1. **Miscarriage, complete** | 196 |
| 1. **Inevitable miscarriage** | 13 |
| 1. **Miscarriage, inevitable** | 13 |
| 1. **Late miscarriage** | 279 |
| 1. **Miscarriage, late** | 279 |
| 1. **Early miscarriage** | 1093 |
| 1. **Miscarriage, early** | 1093 |
| 1. **Silent miscarriage** | 9 |
| 1. Abortion, incomplete | 1315 |
| 1. Incomplete abortion | 1315 |
| 1. Abortions, incomplete | 386 |
| 1. Incomplete, abortions | 386 |
| 1. Abortion, complete | 1890 |
| 1. Complete, abortion | 1890 |
| 1. Abortions, complete | 546 |
| 1. Complete, abortions | 546 |
| 1. Abortion, inevitable | 134 |
| 1. Inevitable, abortion | 134 |
| 1. Abortions, inevitable | 37 |
| 1. Inevitable, abortions | 37 |
| 1. Abortion, tubal | 1176 |
| 1. Abortions, tubal | 277 |
| 1. Tubal, abortion | 1176 |
| 1. Tubal, abortions | 277 |
| 1. Recurrent, miscarriage | 1038 |
| 1. Miscarriage, recurrent | 1038 |
| 1. Recurrent, miscarriages | 720 |
| 1. Miscarriages, recurrent | 720 |
| 1. Septic, miscarriage | 19 |
| 1. Miscarriage, septic | 19 |
| 1. Anembryonic pregnancy | 106 |
| 1. **Fetal death in utero** | 1535 |
| 1. **Intrauterine fetal death** | 3911 |
| 1. **Embryonic demise** | 124 |
| 1. **Stillbirth** | 4843 |
| 1. [#21 or #22 or #23 or #24 or #25 or #26 or #27 or #28 or #29 or #30 or #31 or #32 or #33 or #34 or #35 or #36 or #37 or #38 or #39 or #40 or #41 or #42 or #43 or #44 or #45 or #46 or #47 or #48 or #49 or #50 or #51 or #52 or #53 or #54 or #55 or #56 or #57 or #58 or #59 or #60 or #61 or #62 or #63 or #64 or #65 or #66 or #67 or #68 or #69 or #70 or #71 or #72] | 35260 |
| 1. [#20 and #73] | 2135 |
| 1. Article | 320400 |
| 1. Cohort study | 155308 |
| 1. Case-control study | 136372 |
| 1. Systematic review | 43461 |
| 1. Review | 924279 |
| 1. Retrospective study | 240429 |
| 1. Prospective study | 283919 |
| 1. [#75 or #76 or #77 or #78 or #79 or #80 or #81] | 1814295 |
| 1. Longterm effects | 2365 |
| 1. Long-term effects | 173418 |
| 1. Long-term complications | 90374 |
| 1. Longterm complications | 1368 |
| 1. Pregnancy outcome | 69680 |
| 1. Postoperative complications | 326756 |
| 1. Subfertility | 1854 |
| 1. Sub-fertility | 98 |
| 1. Subsequent fertility | 4036 |
| 1. Secondary subfertility | 113 |
| 1. [#83 or #84 or #85 or #86 or #87 or #88 or #89 or #90 or #91 or #92] | 606222 |
| 1. **[#20 and #73 and #82 and #93]** | **524** |

| **Search terms** | **No of records returned** |
| --- | --- |
| ***For Embase (1974- November 11^th^ 2011)*** |  |
| 1. Caesarean section | 15148 |
| 1. Caesarean sections | 2100 |
| 1. Cesarean section | 54566 |
| 1. Cesarean sections | 4559 |
| 1. Delivery, abdominal | 124308 |
| 1. Deliveries, abdominal | 1229 |
| 1. Abdominal delivery | 124308 |
| 1. Abdominal deliveries | 1229 |
| 1. C-section | 742 |
| 1. C-sections | 209 |
| 1. C section | 165058 |
| 1. C sections | 55776 |
| 1. Postcesarean section | 297 |
| 1. Postcaesarean section | 26 |
| 1. Caesarean delivery | 14377 |
| 1. Cesarean delivery | 53798 |
| 1. Delivery, Caesarean | 14377 |
| 1. Delivery, Cesarean | 53798 |
| 1. Mode of delivery | 18175 |
| 1. [#1 or #2 or #3 or #4 or #5 or #6 or #7 or #8 or #9 or #10 or #11 or #12 or #13 or #14 or #15 or #16 or #17 or #18 or #19] | 385875 |
| 1. Abortion, spontaneous | 24619 |
| 1. Spontaneous, abortion | 24619 |
| 1. Abortions, spontaneous | 5512 |
| 1. Spontaneous, abortions | 5512 |
| 1. Abortion, missed | 1639 |
| 1. Missed, abortion | 1639 |
| 1. Abortions, missed | 312 |
| 1. Missed, abortions | 312 |
| 1. Miscarriage | 22999 |
| 1. Miscarriages | 3449 |
| 1. Spontaneous, miscarriage | 21045 |
| 1. Spontaneous, miscarriages | 2383 |
| 1. Miscarriage, spontaneous | 21045 |
| 1. Miscarriages, spontaneous | 2383 |
| 1. **Incomplete miscarriage** | 284 |
| 1. **Miscarriage, incomplete** | 284 |
| 1. **Complete miscarriage** | 672 |
| 1. **Miscarriage, complete** | 672 |
| 1. **Inevitable miscarriage** | 50 |
| 1. **Miscarriage, inevitable** | 50 |
| 1. **Late miscarriage** | 841 |
| 1. **Miscarriage, late** | 841 |
| 1. **Early miscarriage** | 3595 |
| 1. **Miscarriage, early** | 3595 |
| 1. **Silent miscarriage** | 33 |
| 1. Abortion, incomplete | 1051 |
| 1. Incomplete abortion | 1051 |
| 1. Abortions, incomplete | 298 |
| 1. Incomplete, abortions | 298 |
| 1. Abortion, complete | 2170 |
| 1. Complete, abortion | 2170 |
| 1. Abortions, complete | 543 |
| 1. Complete, abortions | 543 |
| 1. Abortion, inevitable | 156 |
| 1. Inevitable, abortion | 156 |
| 1. Abortions, inevitable | 41 |
| 1. Inevitable, abortions | 41 |
| 1. Abortion, tubal | 911 |
| 1. Abortions, tubal | 221 |
| 1. Tubal, abortion | 911 |
| 1. Tubal, abortions | 221 |
| 1. Recurrent, miscarriage | 3532 |
| 1. Miscarriage, recurrent | 3532 |
| 1. Recurrent, miscarriages | 1157 |
| 1. Miscarriages, recurrent | 1157 |
| 1. Septic, miscarriage | 144 |
| 1. Miscarriage, septic | 144 |
| 1. Anembryonic pregnancy | 122 |
| 1. **Fetal death in utero** | 1751 |
| 1. **Intrauterine fetal death** | 5777 |
| 1. **Embryonic demise** | 735 |
| 1. **Stillbirth** | 8882 |
| 1. [#21 or #22 or #23 or #24 or #25 or #26 or #27 or #28 or #29 or #30 or #31 or #32 or #33 or #34 or #35 or #36 or #37 or #38 or #39 or #40 or #41 or #42 or #43 or #44 or #45 or #46 or #47 or #48 or #49 or #50 or #51 or #52 or #53 or #54 or #55 or #56 or #57 or #58 or #59 or #60 or #61 or #62 or #63 or #64 or #65 or #66 or #67 or #68 or #69 or #70 or #71 or #72] | 45126 |
| 1. [#20 and #73] | 4305 |
| 1. Article | 23846466 |
| 1. Cohort study | 242035 |
| 1. Case-control study | 322084 |
| 1. Systematic review | 92233 |
| 1. Review | 2465399 |
| 1. Retrospective study | 377342 |
| 1. Prospective study | 409335 |
| 1. [#75 or #76 or #77 or #78 or #79 or #80 or #81] | 23885931 |
| 1. Longterm effects | 2357 |
| 1. Long-term effects | 170710 |
| 1. Long-term complications | 105153 |
| 1. Longterm complications | 1219 |
| 1. Pregnancy outcome | 89142 |
| 1. Postoperative complications | 152566 |
| 1. Subfertility | 3466 |
| 1. Sub-fertility | 169 |
| 1. Subsequent fertility | 7409 |
| 1. Secondary subfertility | 193 |
| 1. [#83 or #84 or #85 or #86 or #87 or #88 or #89 or #90 or #91 or #92] | 470588 |
| 1. **[#20 and #73 and #82 and #93]** | **2042** |

| **Search terms** | **No of records returned** |
| --- | --- |
| ***For Web of Knowledge (1945- November 11^th^ 2011)*** |  |
| 1. Caesarean section | 80152 |
| 1. Caesarean sections | 80152 |
| 1. Cesarean section | 80637 |
| 1. Cesarean sections | 80637 |
| 1. Delivery, abdominal | 8935 |
| 1. Deliveries, abdominal | 8935 |
| 1. Abdominal delivery | 8935 |
| 1. Abdominal deliveries | 8935 |
| 1. C-section | 954 |
| 1. C-sections | 954 |
| 1. C section | 167804 |
| 1. C sections | 167804 |
| 1. Postcesarean section | 393 |
| 1. Postcaesarean section | 21 |
| 1. Caesarean delivery | 45513 |
| 1. Cesarean delivery | 45727 |
| 1. Delivery, Caesarean | 45513 |
| 1. Delivery, Cesarean | 45727 |
| 1. Mode of delivery | 19435 |
| 1. [#1 or #2 or #3 or #4 or #5 or #6 or #7 or #8 or #9 or #10 or #11 or #12 or #13 or #14 or #15 or #16 or #17 or #18 or #19] | 274690 |
| 1. Abortion, spontaneous | 37716 |
| 1. Spontaneous, abortion | 37716 |
| 1. Abortions, spontaneous | 37716 |
| 1. Spontaneous, abortions | 37716 |
| 1. Abortion, missed | 1963 |
| 1. Missed, abortion | 1963 |
| 1. Abortions, missed | 1963 |
| 1. Missed, abortions | 1963 |
| 1. Miscarriage | 18453 |
| 1. Miscarriages | 18453 |
| 1. Spontaneous, miscarriage | 7083 |
| 1. Spontaneous, miscarriages | 7083 |
| 1. Miscarriage, spontaneous | 7083 |
| 1. Miscarriages, spontaneous | 7083 |
| 1. **Incomplete miscarriage** | 200 |
| 1. **Miscarriage, incomplete** | 200 |
| 1. **Complete miscarriage** | 634 |
| 1. **Miscarriage, complete** | 634 |
| 1. **Inevitable miscarriage** | 23 |
| 1. **Miscarriage, inevitable** | 23 |
| 1. **Late miscarriage** | 852 |
| 1. **Miscarriage, late** | 852 |
| 1. **Early miscarriage** | 3826 |
| 1. **Miscarriage, early** | 3826 |
| 1. **Silent miscarriage** | 14 |
| 1. Abortion, incomplete | 1573 |
| 1. Incomplete abortion | 1573 |
| 1. Abortions, incomplete | 1573 |
| 1. Incomplete, abortions | 1573 |
| 1. Abortion, complete | 5776 |
| 1. Complete, abortion | 5776 |
| 1. Abortions, complete | 5776 |
| 1. Complete, abortions | 5776 |
| 1. Abortion, inevitable | 165 |
| 1. Inevitable, abortion | 165 |
| 1. Abortions, inevitable | 165 |
| 1. Inevitable, abortions | 165 |
| 1. Abortion, tubal | 1576 |
| 1. Abortions, tubal | 1576 |
| 1. Tubal, abortion | 1576 |
| 1. Tubal, abortions | 1576 |
| 1. Recurrent, miscarriage | 4762 |
| 1. Miscarriage, recurrent | 4762 |
| 1. Recurrent, miscarriages | 4762 |
| 1. Miscarriages, recurrent | 4762 |
| 1. Septic, miscarriage | 28 |
| 1. Miscarriage, septic | 28 |
| 1. Anembryonic pregnancy | 141 |
| 1. **Fetal death in utero** | 3095 |
| 1. **Intrauterine fetal death** | 8290 |
| 1. **Embryonic demise** | 277 |
| 1. **Stillbirth** | 16416 |
| 1. [#21 or #22 or #23 or #24 or #25 or #26 or #27 or #28 or #29 or #30 or #31 or #32 or #33 or #34 or #35 or #36 or #37 or #38 or #39 or #40 or #41 or #42 or #43 or #44 or #45 or #46 or #47 or #48 or #49 or #50 or #51 or #52 or #53 or #54 or #55 or #56 or #57 or #58 or #59 or #60 or #61 or #62 or #63 or #64 or #65 or #66 or #67 or #68 or #69 or #70 or #71 or #72] | 80276 |
| 1. [#20 and #73] | 4828 |
| 1. Article | 1080251 |
| 1. Cohort study | 486755 |
| 1. Case-control study | 642749 |
| 1. Systematic review | 127198 |
| 1. Review | 2640379 |
| 1. Retrospective study | 644339 |
| 1. Prospective study | 760601 |
| 1. [#75 or #76 or #77 or #78 or #79 or #80 or #81] | 5312269 |
| 1. Longterm effects | 7479 |
| 1. Long-term effects | 516063 |
| 1. Long-term complications | 159726 |
| 1. Longterm complications | 2464 |
| 1. Pregnancy outcome | 141336 |
| 1. Postoperative complications | 425799 |
| 1. Subfertility | 5013 |
| 1. Sub-fertility | 183 |
| 1. Subsequent fertility | 10488 |
| 1. Secondary subfertility | 170 |
| 1. [#83 or #84 or #85 or #86 or #87 or #88 or #89 or #90 or #91 or #92] | 1162002 |
| 1. **[#20 and #73 and #82 and #93]** | **959** |

| **Search terms** | **No of records returned** |
| --- | --- |
| ***For Scopus (1960-Novmeber 11^th^ 2011 )*** |  |
| 1. Caesarean section | 25580 |
| 1. Caesarean sections | 14060 |
| 1. Cesarean section | 55783 |
| 1. Cesarean sections | 55783 |
| 1. Delivery, abdominal | 6841 |
| 1. Deliveries, abdominal | 6841 |
| 1. Abdominal delivery | 6841 |
| 1. Abdominal deliveries | 6841 |
| 1. C-section | 992 |
| 1. C-sections | 992 |
| 1. C section | 202204 |
| 1. C sections | 202204 |
| 1. Postcesarean section | 308 |
| 1. Postcaesarean section | 24 |
| 1. Caesarean delivery | 7388 |
| 1. Cesarean delivery | 26447 |
| 1. Delivery, Caesarean | 7388 |
| 1. Delivery, Cesarean | 26447 |
| 1. Mode of delivery | 12741 |
| 1. [#1 or #2 or #3 or #4 or #5 or #6 or #7 or #8 or #9 or #10 or #11 or #12 or #13 or #14 or #15 or #16 or #17 or #18 or #19] | 43117732 |
| 1. Abortion, spontaneous | 27243 |
| 1. Spontaneous, abortion | 27243 |
| 1. Abortions, spontaneous | 27243 |
| 1. Spontaneous, abortions | 27243 |
| 1. Abortion, missed | 1831 |
| 1. Missed, abortion | 1831 |
| 1. Abortions, missed | 1831 |
| 1. Missed, abortions | 1831 |
| 1. Miscarriage | 8661 |
| 1. Miscarriages | 8661 |
| 1. Spontaneous, miscarriage | 5535 |
| 1. Spontaneous, miscarriages | 5535 |
| 1. Miscarriage, spontaneous | 5535 |
| 1. Miscarriages, spontaneous | 5535 |
| 1. **Incomplete miscarriage** | 165 |
| 1. **Miscarriage, incomplete** | 165 |
| 1. **Complete miscarriage** | 335 |
| 1. **Miscarriage, complete** | 335 |
| 1. **Inevitable miscarriage** | 26 |
| 1. **Miscarriage, inevitable** | 26 |
| 1. **Late miscarriage** | 776 |
| 1. **Miscarriage, late** | 776 |
| 1. **Early miscarriage** | 1822 |
| 1. **Miscarriage, early** | 1822 |
| 1. **Silent miscarriage** | 14 |
| 1. Abortion, incomplete | 1579 |
| 1. Incomplete abortion | 1579 |
| 1. Abortions, incomplete | 1579 |
| 1. Incomplete, abortions | 1579 |
| 1. Abortion, complete | 2596 |
| 1. Complete, abortion | 2596 |
| 1. Abortions, complete | 2596 |
| 1. Complete, abortions | 2596 |
| 1. Abortion, inevitable | 196 |
| 1. Inevitable, abortion | 196 |
| 1. Abortions, inevitable | 196 |
| 1. Inevitable, abortions | 196 |
| 1. Abortion, tubal | 1543 |
| 1. Abortions, tubal | 1543 |
| 1. Tubal, abortion | 1543 |
| 1. Tubal, abortions | 1543 |
| 1. Recurrent, miscarriage | 2027 |
| 1. Miscarriage, recurrent | 2027 |
| 1. Recurrent, miscarriages | 2027 |
| 1. Miscarriages, recurrent | 2027 |
| 1. Septic, miscarriage | 33 |
| 1. Miscarriage, septic | 33 |
| 1. Anembryonic pregnancy | 119 |
| 1. **Fetal death in utero** | 2020 |
| 1. **Intrauterine fetal death** | 6118 |
| 1. **Embryonic demise** | 139 |
| 1. **Stillbirth** | 10725 |
| 1. [#21 or #22 or #23 or #24 or #25 or #26 or #27 or #28 or #29 or #30 or #31 or #32 or #33 or #34 or #35 or #36 or #37 or #38 or #39 or #40 or #41 or #42 or #43 or #44 or #45 or #46 or #47 or #48 or #49 or #50 or #51 or #52 or #53 or #54 or #55 or #56 or #57 or #58 or #59 or #60 or #61 or #62 or #63 or #64 or #65 or #66 or #67 or #68 or #69 or #70 or #71 or #72] | 33285959 |
| 1. [#20 and #73] | 41968 |
| 1. Article | 16621877 |
| 1. Cohort study | 258582 |
| 1. Case-control study | 382210 |
| 1. Systematic review | 88201 |
| 1. Review | 2783060 |
| 1. Retrospective study | 492821 |
| 1. Prospective study | 459440 |
| 1. [#75 or #76 or #77 or #78 or #79 or #80 or #81] | 9640119 |
| 1. Longterm effects | 4318 |
| 1. Long-term effects | 254248 |
| 1. Long-term complications | 75224 |
| 1. Longterm complications | 1681 |
| 1. Pregnancy outcome | 99236 |
| 1. Postoperative complications | 411735 |
| 1. Subfertility | 3241 |
| 1. Sub-fertility | 147 |
| 1. Subsequent fertility | 4438 |
| 1. Secondary subfertility | 166 |
| 1. [#83 or #84 or #85 or #86 or #87 or #88 or #89 or #90 or #91 or #92] | 11704060 |
| 1. **[#20 and #73 and #82 and #93]** | **455** |

| **Search terms** | **No of records returned** |
| --- | --- |
| ***For Cochrane (1993-November 11^th^ 2011 )*** |  |
| 1. Caesarean section | 1568 |
| 1. Caesarean sections | 1568 |
| 1. Cesarean section | 3346 |
| 1. Cesarean sections | 3346 |
| 1. Delivery, abdominal | 241 |
| 1. Deliveries, abdominal | 241 |
| 1. Abdominal delivery | 241 |
| 1. Abdominal deliveries | 241 |
| 1. C-section | 32 |
| 1. C-sections | 32 |
| 1. C section | 458 |
| 1. C sections | 458 |
| 1. Postcesarean section | 29 |
| 1. Postcaesarean section | 3 |
| 1. Caesarean delivery | 595 |
| 1. Cesarean delivery | 417 |
| 1. Delivery, Caesarean | 595 |
| 1. Delivery, Cesarean | 1417 |
| 1. Mode of delivery | 684 |
| 1. [#1 or #2 or #3 or #4 or #5 or #6 or #7 or #8 or #9 or #10 or #11 or #12 or #13 or #14 or #15 or #16 or #17 or #18 or #19] | 250128 |
| 1. Abortion, spontaneous | 671 |
| 1. Spontaneous, abortion | 671 |
| 1. Abortions, spontaneous | 671 |
| 1. Spontaneous, abortions | 671 |
| 1. Abortion, missed | 243 |
| 1. Missed, abortion | 243 |
| 1. Abortions, missed | 243 |
| 1. Missed, abortions | 243 |
| 1. Miscarriage | 728 |
| 1. Miscarriages | 728 |
| 1. Spontaneous, miscarriage | 293 |
| 1. Spontaneous, miscarriages | 293 |
| 1. Miscarriage, spontaneous | 293 |
| 1. Miscarriages, spontaneous | 293 |
| 1. **Incomplete miscarriage** | 171 |
| 1. **Miscarriage, incomplete** | 171 |
| 1. **Complete miscarriage** | 231 |
| 1. **Miscarriage, complete** | 231 |
| 1. **Inevitable miscarriage** | 14 |
| 1. **Miscarriage, inevitable** | 14 |
| 1. **Late miscarriage** | 180 |
| 1. **Miscarriage, late** | 180 |
| 1. **Early miscarriage** | 287 |
| 1. **Miscarriage, early** | 287 |
| 1. **Silent miscarriage** | 5 |
| 1. Abortion, incomplete | 270 |
| 1. Incomplete abortion | 270 |
| 1. Abortions, incomplete | 270 |
| 1. Incomplete, abortions | 270 |
| 1. Abortion, complete | 478 |
| 1. Complete, abortion | 478 |
| 1. Abortions, complete | 478 |
| 1. Complete, abortions | 478 |
| 1. Abortion, inevitable | 19 |
| 1. Inevitable, abortion | 19 |
| 1. Abortions, inevitable | 19 |
| 1. Inevitable, abortions | 19 |
| 1. Abortion, tubal | 68 |
| 1. Abortions, tubal | 68 |
| 1. Tubal, abortion | 68 |
| 1. Tubal, abortions | 68 |
| 1. Recurrent, miscarriage | 143 |
| 1. Miscarriage, recurrent | 143 |
| 1. Recurrent, miscarriages | 143 |
| 1. Miscarriages, recurrent | 143 |
| 1. Septic, miscarriage | 0 |
| 1. Miscarriage, septic | 0 |
| 1. Anembryonic pregnancy | 10 |
| 1. **Fetal death in utero** | 133 |
| 1. **Intrauterine fetal death** | 264 |
| 1. **Embryonic demise** | 5 |
| 1. **Stillbirth** | 367 |
| 1. [#21 or #22 or #23 or #24 or #25 or #26 or #27 or #28 or #29 or #30 or #31 or #32 or #33 or #34 or #35 or #36 or #37 or #38 or #39 or #40 or #41 or #42 or #43 or #44 or #45 or #46 or #47 or #48 or #49 or #50 or #51 or #52 or #53 or #54 or #55 or #56 or #57 or #58 or #59 or #60 or #61 or #62 or #63 or #64 or #65 or #66 or #67 or #68 or #69 or #70 or #71 or #72] | 1999 |
| 1. [#20 and #73] | 1269 |
| 1. Article | 721290 |
| 1. Cohort study | 17428 |
| 1. Case-control study | 53393 |
| 1. Systematic review | 29157 |
| 1. Review | 111932 |
| 1. Retrospective study | 11233 |
| 1. Prospective study | 92722 |
| 1. [#75 or #76 or #77 or #78 or #79 or #80 or #81] | 721290 |
| 1. Longterm effects | 6418 |
| 1. Long-term effects | 28634 |
| 1. Long-term complications | 8970 |
| 1. Longterm complications | 1492 |
| 1. Pregnancy outcome | 4 |
| 1. Postoperative complications | 20659 |
| 1. Subfertility | 352 |
| 1. Sub-fertility | 28 |
| 1. Subsequent fertility | 296 |
| 1. Secondary subfertility | 213 |
| 1. [#83 or #84 or #85 or #86 or #87 or #88 or #89 or #90 or #91 or #92] | 50359 |
| 1. **[#20 and #73 and #82 and #93]** | **445** |
